# 2-Amino-6-(piperidin-1-yl)-4-*p*-tolyl­pyridine-3,5-dicarbo­nitrile

**DOI:** 10.1107/S1600536813030845

**Published:** 2013-11-23

**Authors:** S. Antony Inglebert, Jayabal Kamalraja, K. Sethusankar, Gnanasambandam Vasuki

**Affiliations:** a Sri Ram Engineering College, Chennai 602 024, India; b Department of Chemistry, Pondichery University, Pondichery 605 014, India; c Department of Physics, RKM Vivekananda College (Autonomous), Chennai 600 004, India

## Abstract

In the title compound, C_19_H_19_N_5_, the piperidine ring adopts a chair conformation. The pyridine ring is essentially planar, with a maximum deviation of 0.039 (2) Å for a C atom substituted with a carbonitrile group. The mean plane of the central pyridine ring makes the dihedral angles of 37.90 (14) and 56.10 (12)° with the piperidine and benzene rings, respectively. In the crystal, mol­ecules are linked *via* N—H⋯N and C—H⋯N hydrogen bonds, forming chains along [101], and enclosing *R*_2_^2^(17) ring motifs. The chains are linked by further C—H⋯N hydrogen bonds, forming two-dimensional networks lying parallel to (10-1), and enclosing inversion dimers with *R*_2_^2^(20) ring motifs.

## Related literature

For background to pyridine derivatives and their biological activity, see: Chaubey & Pandeya (2011 ▶). For puckering parameters, see: Cremer & Pople (1975 ▶). For graph-set notation, see: Bernstein *et al.* (1995 ▶). For a related structure, see: Inglebert *et al.* (2011 ▶).
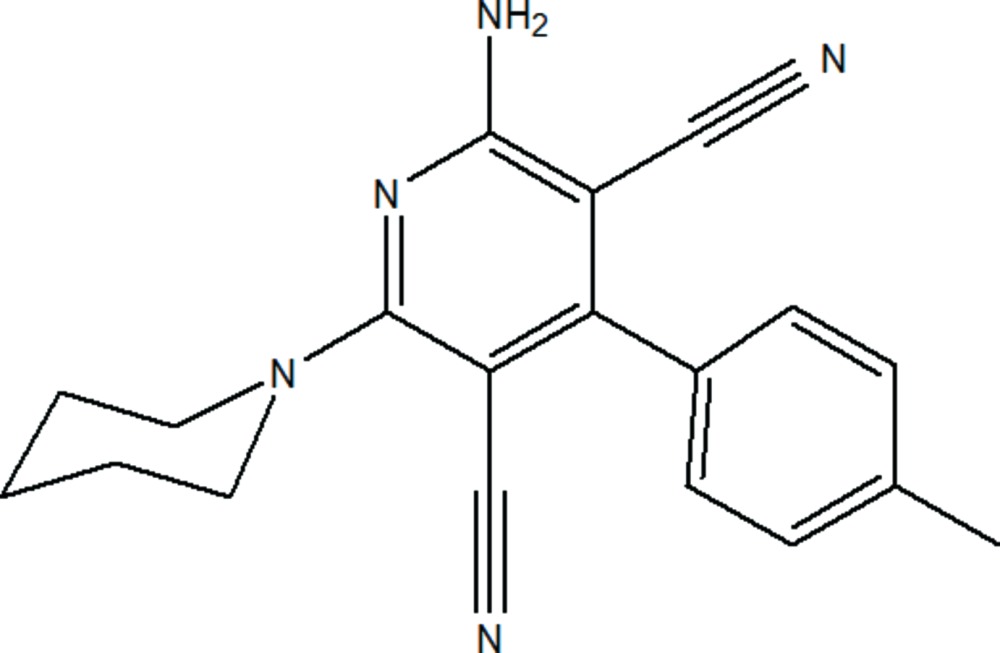


## Experimental

### 

#### Crystal data

C_19_H_19_N_5_
*M* *_r_* = 317.39
Monoclinic, 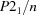

*a* = 14.8695 (12) Å
*b* = 7.7350 (6) Å
*c* = 15.2791 (13) Å
β = 107.196 (8)°
*V* = 1678.8 (2) Å^3^
*Z* = 4
Mo *K*α radiation
μ = 0.08 mm^−1^
*T* = 295 K
0.37 × 0.30 × 0.25 mm

#### Data collection

Bruker Kappa APEXII CCD diffractometer
Absorption correction: multi-scan (*SADABS*; Bruker, 2008 ▶) *T*_min_ = 0.972, *T*_max_ = 0.981
7726 measured reflections
3705 independent reflections
1346 reflections with *I* > 2σ(*I*)
*R*_int_ = 0.056

#### Refinement

*R*[*F* ^2^ > 2σ(*F* ^2^)] = 0.060
*wR*(*F* ^2^) = 0.148
*S* = 0.77
3705 reflections
218 parameters
H-atom parameters constrained
Δρ_max_ = 0.18 e Å^−3^
Δρ_min_ = −0.29 e Å^−3^

### 

Data collection: *APEX2* (Bruker, 2008 ▶); cell refinement: *SAINT* (Bruker, 2008 ▶); data reduction: *SAINT*; program(s) used to solve structure: *SHELXS97* (Sheldrick, 2008 ▶); program(s) used to refine structure: *SHELXL97* (Sheldrick, 2008 ▶); molecular graphics: *ORTEP-3* for Windows (Farrugia, 2012 ▶) and *Mercury* (Macrae *et al.*, 2008 ▶); software used to prepare material for publication: *SHELXL97* and *PLATON* (Spek, 2009 ▶).

## Supplementary Material

Crystal structure: contains datablock(s) global, I. DOI: 10.1107/S1600536813030845/rk2416sup1.cif

Structure factors: contains datablock(s) I. DOI: 10.1107/S1600536813030845/rk2416Isup2.hkl

Supplementary material file. DOI: 10.1107/S1600536813030845/rk2416Isup3.cml

Additional supplementary materials:  crystallographic information; 3D view; checkCIF report

## Figures and Tables

**Table 1:** Hydrogen-bond geometry (Å, °)

*D*—H⋯*A*	*D*—H	H⋯*A*	*D*⋯*A*	*D*—H⋯*A*
N5—H5*C*⋯N3^i^	0.86	2.18	2.999 (4)	160
C2—H2*B*⋯N4^ii^	0.97	2.62	3.509 (4)	153
C19—H19*A*⋯N4^iii^	0.96	2.61	3.509 (4)	156

Symmetry codes: (i) 
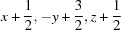
; (ii) 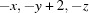; (iii) 
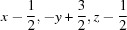
.

**Fig. 1.**
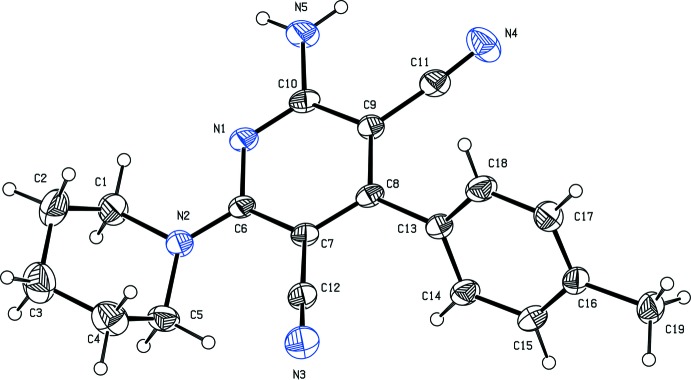
A view of the molecular structure, showing the atom numbering scheme. Displacement ellipsoids are drawn at the 30% probability level. H atoms are presented as a small spheres of arbitrary radius.

**Fig. 2.**
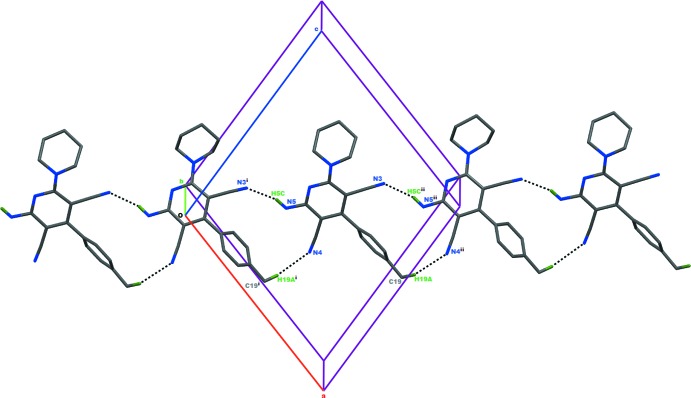
The packing structure of the title compound shows two intermolecular C—H···N and N—H···N hydrogen bond to generate R22(17) motif. H atoms have been omited for clarity.

**Fig. 3.**
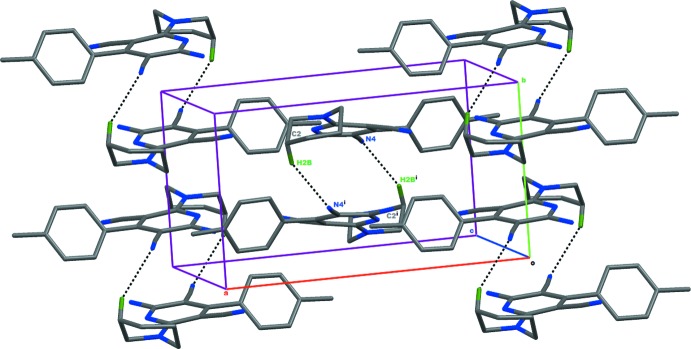
The packing structure of the title compound shows another pair of intermolecular C—H···N hydrogen bonds to form inversion dimer. H atoms have been omited for clarity.

## supplementary crystallographic information

### 1. Comment

Pyridine ring system is widely distributed in nature, especially in plant kingdom. Many important alkaloids atropine from Deadly nightshade (*Atropa belladonna*) contains saturated pyridine nucleus. The pyridine is found to have a large number of biological activities those including antiviral, anticancer, antimicrobial, antidiabetic and antitubercular. Pyridine is also a very active neutraceutical found in the form of vitamin B3 (Chaubey & Pandeya, 2011).

The cyano groups are flipped to different sides of the pyridine plane with atoms C12 & N3 showing deviations of 0.3416 (1)Å and 0.6489 (1)Å, while atoms C11 & N4 are bent out of the pyridine plane by -0.0866 (1)Å and -0.1408 (1)Å, respectively. The pyridine ring (N1/C6–C10) forms dihedral angles of 37.90 (14)° and 56.10 (12)° with piperidine (N2/C1–C5) and phenyl ring (C13–C18).

The pyridine ring is essentially planar with a maximum deviation of -0.039 (2) Å for C7 atom. The piperidine ring adopts a *chair* conformation [puckering parameters (Cremer & Pople, 1975): Q = 0.579 (3)Å, θ = 172.5 (4)° and φ = 152 (3)°]. The amino group lies in the pyridine ring. The title compound exhibits the structural similarities with the reported related structure (Inglebert *et al.*, 2011).

In the crystal, molecules are connected through the intermolecular C—H···N and N—H···N hydrogen bonds, generating a *R*_2_^2^(17) (Bernstein *et al.*, 1995) motif and also chain along *a* axis. In addition, another pair of intermolecular, C—H···N hydrogen bond link neighbouring molecules, forming an inversion dimer and generate a *R*_2_^2^(20) ring motif (Table 1).

### 2. Experimental

Initially a mixture of 4–methylbenzaldehyde (2 mmol, 0.24 g), malononitrile (3 mmol, 0.198 g), piperidine (1.5 mmol, 0.128 g) and was stirred without any solvent at room temperature. A solid appeared immediately which was dissolved in a minimum amount (3 ml) of ethanol and the solution was refluxed until completion of the reaction (monitered by *TLC*). The reaction mixture was cooled. Ethanol was evaporated under reduced pressure and the residue was extracted with dichloromethane (3×10ml). Evaporation of solvent left the crude solid which was subjected to silica gel column chromatography [25 : 75 / ethyl acetate : hexane] and the product was recrystallized from dichloromethane.

### 3. Refinement

H atoms were placed in idealized positions and allowed to ride on the parent atoms,with C—H bond lengths fixed to 0.93Å (aromatic H), 0.96Å (methyl H),0.97Å (methylene H), 0.86Å (N—H) and *U*_iso_(H) = 1.2–1.5*U*_eq_(C,N).

### Crystal data

**Table:**

C_19_H_19_N_5_	*F*(000) = 672
*M**_r_* = 317.39	*D*_x_ = 1.256 Mg m^−^^3^
Monoclinic, *P*2_1_/*n*	Mo *K*α radiation, λ = 0.71073 Å
Hall symbol: -p 2yn	Cell parameters from 3705 reflections
*a* = 14.8695 (12) Å	θ = 2.8–27.5°
*b* = 7.7350 (6) Å	µ = 0.08 mm^−^^1^
*c* = 15.2791 (13) Å	*T* = 295 K
β = 107.196 (8)°	Block, colourless
*V* = 1678.8 (2) Å^3^	0.37 × 0.30 × 0.25 mm
*Z* = 4	

### Data collection

**Table:**

Bruker Kappa APEXII CCD diffractometer	3705 independent reflections
Radiation source: fine–focus sealed tube	1346 reflections with *I* > 2σ(*I*)
Graphite monochromator	*R*_int_ = 0.056
ω and φ scans	θ_max_ = 27.5°, θ_min_ = 2.8°
Absorption correction: multi-scan (*SADABS*; Bruker, 2008)	*h* = −19→8
*T*_min_ = 0.972, *T*_max_ = 0.981	*k* = −10→9
7726 measured reflections	*l* = −19→19

### Refinement

**Table:**

Refinement on *F*^2^	Primary atom site location: structure-invariant direct methods
Least-squares matrix: full	Secondary atom site location: difference Fourier map
*R*[*F*^2^ > 2σ(*F*^2^)] = 0.060	Hydrogen site location: inferred from neighbouring sites
*wR*(*F*^2^) = 0.148	H-atom parameters constrained
*S* = 0.77	*w* = 1/[σ^2^(*F*_o_^2^) + (0.068*P*)^2^ + 0.0452*P*] where *P* = (*F*_o_^2^ + 2*F*_c_^2^)/3
3705 reflections	(Δ/σ)_max_ < 0.001
218 parameters	Δρ_max_ = 0.18 e Å^−^^3^
0 restraints	Δρ_min_ = −0.29 e Å^−^^3^

### Special details

**Table:**

Geometry. All s.u.'s (except the s.u. in the dihedral angle between two l.s. planes) are estimated using the full covariance matrix. The cell s.u.'s are taken into account individually in the estimation of s.u.'s in distances, angles and torsion angles; correlations between s.u.'s in cell parameters are only used when they are defined by crystal symmetry. An approximate (isotropic) treatment of cell s.u.'s is used for estimating s.u.'s involving l.s. planes.
Refinement. Refinement of *F*^2^ against ALL reflections. The weighted *R*–factor w*R* and goodness of fit *S* are based on *F*^2^, conventional *R*–factors *R* are based on *F*, with *F* set to zero for negative *F*^2^. The threshold expression of *F*^2^ > 2σ(*F*^2^) is used only for calculating *R*–factors(gt) etc. and is not relevant to the choice of reflections for refinement. *R*–factors based on *F*^2^ are statistically about twice as large as those based on *F*, and *R*–factors based on ALL data will be even larger.

### Fractional atomic coordinates and isotropic or equivalent isotropic displacement parameters (Å^2^)

**Table:**

	*x*	*y*	*z*	*U*_iso_*/*U*_eq_	
C1	0.18652 (19)	0.6347 (4)	−0.1252 (2)	0.0608 (9)	
H1A	0.2077	0.5189	−0.1334	0.073*	
H1B	0.2152	0.6684	−0.0619	0.073*	
C2	0.2160 (2)	0.7592 (4)	−0.1883 (3)	0.0738 (10)	
H2A	0.2834	0.7499	−0.1776	0.089*	
H2B	0.2025	0.8763	−0.1733	0.089*	
C3	0.1661 (3)	0.7255 (4)	−0.2895 (3)	0.0804 (11)	
H3A	0.1796	0.8190	−0.3261	0.097*	
H3B	0.1895	0.6189	−0.3083	0.097*	
C4	0.0606 (2)	0.7120 (4)	−0.3062 (2)	0.0694 (9)	
H4A	0.0355	0.8241	−0.2971	0.083*	
H4B	0.0305	0.6762	−0.3689	0.083*	
C5	0.0396 (2)	0.5832 (4)	−0.24158 (19)	0.0541 (8)	
H5A	−0.0280	0.5735	−0.2534	0.065*	
H5B	0.0636	0.4707	−0.2516	0.065*	
C6	0.03918 (17)	0.6941 (3)	−0.08729 (18)	0.0377 (6)	
C7	−0.06080 (18)	0.7199 (3)	−0.10909 (17)	0.0393 (7)	
C8	−0.09990 (17)	0.7577 (3)	−0.03913 (17)	0.0371 (6)	
C9	−0.04006 (17)	0.7845 (3)	0.04943 (17)	0.0390 (6)	
C10	0.05840 (18)	0.7707 (3)	0.06310 (18)	0.0423 (7)	
C11	−0.07316 (18)	0.8218 (4)	0.1260 (2)	0.0469 (7)	
C12	−0.1223 (2)	0.7317 (4)	−0.2007 (2)	0.0501 (7)	
C13	−0.20408 (17)	0.7636 (3)	−0.05558 (17)	0.0389 (7)	
C14	−0.25728 (18)	0.6183 (3)	−0.09053 (18)	0.0480 (7)	
H14	−0.2291	0.5234	−0.1090	0.058*	
C15	−0.35192 (19)	0.6144 (4)	−0.09781 (19)	0.0503 (8)	
H15	−0.3865	0.5158	−0.1212	0.060*	
C16	−0.39660 (18)	0.7517 (4)	−0.07161 (18)	0.0457 (7)	
C17	−0.34374 (19)	0.8981 (4)	−0.04005 (19)	0.0530 (8)	
H17	−0.3729	0.9948	−0.0245	0.064*	
C18	−0.24811 (19)	0.9038 (4)	−0.03101 (19)	0.0510 (8)	
H18	−0.2137	1.0029	−0.0082	0.061*	
C19	−0.49842 (18)	0.7409 (4)	−0.0753 (2)	0.0621 (9)	
H19A	−0.5336	0.6874	−0.1317	0.093*	
H19B	−0.5224	0.8552	−0.0719	0.093*	
H19C	−0.5044	0.6734	−0.0246	0.093*	
N1	0.09533 (14)	0.7257 (3)	−0.00185 (15)	0.0435 (6)	
N2	0.08305 (15)	0.6368 (3)	−0.14634 (15)	0.0494 (6)	
N3	−0.17316 (17)	0.7510 (3)	−0.27279 (18)	0.0718 (8)	
N4	−0.09375 (18)	0.8519 (3)	0.19069 (18)	0.0690 (8)	
N5	0.11881 (16)	0.8013 (3)	0.14709 (15)	0.0639 (7)	
H5C	0.1785	0.7910	0.1561	0.077*	
H5D	0.0976	0.8310	0.1915	0.077*	

### Atomic displacement parameters (Å^2^)

**Table:**

	*U*^11^	*U*^22^	*U*^33^	*U*^12^	*U*^13^	*U*^23^
C1	0.0354 (18)	0.091 (2)	0.051 (2)	0.0125 (16)	0.0047 (15)	−0.0054 (17)
C2	0.0436 (19)	0.088 (2)	0.091 (3)	−0.0040 (17)	0.0231 (19)	−0.004 (2)
C3	0.081 (3)	0.096 (3)	0.072 (3)	0.000 (2)	0.034 (2)	0.015 (2)
C4	0.073 (2)	0.084 (2)	0.045 (2)	0.0123 (18)	0.0078 (18)	0.0084 (17)
C5	0.0489 (18)	0.0681 (19)	0.0394 (18)	0.0019 (15)	0.0037 (14)	−0.0112 (15)
C6	0.0289 (15)	0.0466 (15)	0.0312 (16)	−0.0013 (12)	−0.0008 (12)	−0.0032 (13)
C7	0.0308 (14)	0.0550 (17)	0.0243 (15)	−0.0002 (13)	−0.0041 (12)	0.0013 (12)
C8	0.0289 (14)	0.0418 (15)	0.0321 (15)	−0.0006 (12)	−0.0043 (12)	0.0029 (12)
C9	0.0305 (14)	0.0521 (16)	0.0287 (16)	0.0030 (13)	0.0001 (12)	0.0008 (13)
C10	0.0328 (15)	0.0516 (16)	0.0308 (16)	0.0012 (13)	−0.0086 (13)	0.0005 (13)
C11	0.0366 (17)	0.0588 (17)	0.0363 (18)	0.0075 (14)	−0.0032 (14)	0.0022 (15)
C12	0.0346 (16)	0.073 (2)	0.0365 (17)	−0.0008 (15)	0.0006 (13)	−0.0015 (15)
C13	0.0291 (14)	0.0505 (16)	0.0302 (15)	0.0044 (13)	−0.0018 (11)	0.0026 (13)
C14	0.0348 (17)	0.0562 (18)	0.0464 (19)	−0.0003 (15)	0.0018 (14)	−0.0069 (14)
C15	0.0346 (17)	0.0627 (19)	0.0467 (19)	−0.0072 (15)	0.0013 (14)	−0.0071 (15)
C16	0.0298 (15)	0.0728 (19)	0.0286 (16)	−0.0015 (16)	−0.0005 (12)	0.0051 (15)
C17	0.0378 (18)	0.069 (2)	0.048 (2)	0.0125 (16)	0.0066 (14)	−0.0023 (15)
C18	0.0380 (17)	0.0588 (18)	0.0479 (19)	−0.0024 (15)	−0.0002 (14)	−0.0071 (15)
C19	0.0348 (16)	0.103 (2)	0.045 (2)	−0.0013 (16)	0.0072 (14)	−0.0007 (17)
N1	0.0292 (12)	0.0603 (14)	0.0322 (14)	0.0019 (11)	−0.0043 (10)	−0.0048 (11)
N2	0.0321 (13)	0.0779 (16)	0.0332 (14)	0.0014 (12)	0.0019 (11)	−0.0058 (12)
N3	0.0418 (15)	0.122 (2)	0.0382 (16)	0.0048 (15)	−0.0092 (12)	0.0035 (15)
N4	0.069 (2)	0.095 (2)	0.0407 (17)	0.0215 (15)	0.0134 (15)	0.0021 (15)
N5	0.0350 (13)	0.1071 (19)	0.0366 (15)	0.0111 (13)	−0.0094 (11)	−0.0156 (14)

### Geometric parameters (Å, º)

**Table:**

C1—N2	1.476 (3)	C9—C10	1.420 (3)
C1—C2	1.516 (4)	C9—C11	1.426 (4)
C1—H1A	0.9700	C10—N1	1.315 (3)
C1—H1B	0.9700	C10—N5	1.352 (3)
C2—C3	1.528 (4)	C11—N4	1.142 (3)
C2—H2A	0.9700	C12—N3	1.147 (3)
C2—H2B	0.9700	C13—C18	1.375 (3)
C3—C4	1.517 (4)	C13—C14	1.388 (3)
C3—H3A	0.9700	C14—C15	1.379 (3)
C3—H3B	0.9700	C14—H14	0.9300
C4—C5	1.498 (4)	C15—C16	1.374 (4)
C4—H4A	0.9700	C15—H15	0.9300
C4—H4B	0.9700	C16—C17	1.381 (4)
C5—N2	1.467 (3)	C16—C19	1.501 (4)
C5—H5A	0.9700	C17—C18	1.388 (4)
C5—H5B	0.9700	C17—H17	0.9300
C6—N2	1.336 (3)	C18—H18	0.9300
C6—N1	1.348 (3)	C19—H19A	0.9600
C6—C7	1.438 (3)	C19—H19B	0.9600
C7—C8	1.390 (3)	C19—H19C	0.9600
C7—C12	1.431 (4)	N5—H5C	0.8600
C8—C9	1.398 (3)	N5—H5D	0.8600
C8—C13	1.495 (3)		
			
N2—C1—C2	109.4 (2)	C8—C9—C10	117.7 (2)
N2—C1—H1A	109.8	C8—C9—C11	123.3 (2)
C2—C1—H1A	109.8	C10—C9—C11	119.0 (2)
N2—C1—H1B	109.8	N1—C10—N5	117.0 (2)
C2—C1—H1B	109.8	N1—C10—C9	123.4 (2)
H1A—C1—H1B	108.2	N5—C10—C9	119.5 (3)
C1—C2—C3	113.0 (3)	N4—C11—C9	175.6 (3)
C1—C2—H2A	109.0	N3—C12—C7	175.8 (3)
C3—C2—H2A	109.0	C18—C13—C14	118.7 (2)
C1—C2—H2B	109.0	C18—C13—C8	122.1 (2)
C3—C2—H2B	109.0	C14—C13—C8	119.0 (2)
H2A—C2—H2B	107.8	C15—C14—C13	120.1 (3)
C4—C3—C2	110.4 (3)	C15—C14—H14	120.0
C4—C3—H3A	109.6	C13—C14—H14	120.0
C2—C3—H3A	109.6	C16—C15—C14	122.0 (3)
C4—C3—H3B	109.6	C16—C15—H15	119.0
C2—C3—H3B	109.6	C14—C15—H15	119.0
H3A—C3—H3B	108.1	C15—C16—C17	117.5 (2)
C5—C4—C3	110.0 (3)	C15—C16—C19	121.0 (3)
C5—C4—H4A	109.7	C17—C16—C19	121.5 (3)
C3—C4—H4A	109.7	C16—C17—C18	121.4 (3)
C5—C4—H4B	109.7	C16—C17—H17	119.3
C3—C4—H4B	109.7	C18—C17—H17	119.3
H4A—C4—H4B	108.2	C13—C18—C17	120.3 (3)
N2—C5—C4	110.5 (2)	C13—C18—H18	119.9
N2—C5—H5A	109.6	C17—C18—H18	119.9
C4—C5—H5A	109.6	C16—C19—H19A	109.5
N2—C5—H5B	109.6	C16—C19—H19B	109.5
C4—C5—H5B	109.6	H19A—C19—H19B	109.5
H5A—C5—H5B	108.1	C16—C19—H19C	109.5
N2—C6—N1	115.3 (2)	H19A—C19—H19C	109.5
N2—C6—C7	124.6 (2)	H19B—C19—H19C	109.5
N1—C6—C7	120.1 (2)	C10—N1—C6	120.1 (2)
C8—C7—C12	116.6 (2)	C6—N2—C5	127.2 (2)
C8—C7—C6	119.3 (2)	C6—N2—C1	122.8 (2)
C12—C7—C6	123.6 (3)	C5—N2—C1	109.8 (2)
C7—C8—C9	119.0 (2)	C10—N5—H5C	120.0
C7—C8—C13	121.7 (2)	C10—N5—H5D	120.0
C9—C8—C13	119.3 (2)	H5C—N5—H5D	120.0
			
N2—C1—C2—C3	−53.8 (3)	C9—C8—C13—C14	−120.1 (3)
C1—C2—C3—C4	50.1 (4)	C18—C13—C14—C15	−1.7 (4)
C2—C3—C4—C5	−52.2 (4)	C8—C13—C14—C15	173.6 (2)
C3—C4—C5—N2	60.4 (3)	C13—C14—C15—C16	0.2 (4)
N2—C6—C7—C8	171.4 (2)	C14—C15—C16—C17	2.1 (4)
N1—C6—C7—C8	−7.6 (4)	C14—C15—C16—C19	−176.6 (3)
N2—C6—C7—C12	−17.2 (4)	C15—C16—C17—C18	−2.9 (4)
N1—C6—C7—C12	163.8 (2)	C19—C16—C17—C18	175.7 (3)
C12—C7—C8—C9	−166.5 (2)	C14—C13—C18—C17	0.8 (4)
C6—C7—C8—C9	5.6 (4)	C8—C13—C18—C17	−174.3 (3)
C12—C7—C8—C13	15.9 (4)	C16—C17—C18—C13	1.5 (4)
C6—C7—C8—C13	−172.1 (2)	N5—C10—N1—C6	−179.7 (2)
C7—C8—C9—C10	−0.3 (3)	C9—C10—N1—C6	1.7 (4)
C13—C8—C9—C10	177.4 (2)	N2—C6—N1—C10	−175.2 (2)
C7—C8—C9—C11	−179.2 (2)	C7—C6—N1—C10	3.9 (4)
C13—C8—C9—C11	−1.5 (4)	N1—C6—N2—C5	176.4 (2)
C8—C9—C10—N1	−3.6 (4)	C7—C6—N2—C5	−2.6 (4)
C11—C9—C10—N1	175.4 (2)	N1—C6—N2—C1	−9.6 (4)
C8—C9—C10—N5	177.9 (2)	C7—C6—N2—C1	171.4 (2)
C11—C9—C10—N5	−3.1 (4)	C4—C5—N2—C6	109.7 (3)
C7—C8—C13—C18	−127.4 (3)	C4—C5—N2—C1	−64.9 (3)
C9—C8—C13—C18	55.0 (3)	C2—C1—N2—C6	−114.6 (3)
C7—C8—C13—C14	57.5 (3)	C2—C1—N2—C5	60.3 (3)

### Hydrogen-bond geometry (Å, º)

**Table:**

*D*—H···*A*	*D*—H	H···*A*	*D*···*A*	*D*—H···*A*
N5—H5*C*···N3^i^	0.86	2.18	2.999 (4)	160
C2—H2*B*···N4^ii^	0.97	2.62	3.509 (4)	153
C19—H19*A*···N4^iii^	0.96	2.61	3.509 (4)	156

Symmetry codes: (i) *x*+1/2, −*y*+3/2, *z*+1/2; (ii) −*x*, −*y*+2, −*z*; (iii) *x*−1/2, −*y*+3/2, *z*−1/2.
